# Transobturator vaginal tape in comparison to tension-free vaginal tape: A prospective trial with a minimum 12 months follow-up

**DOI:** 10.4103/0970-1591.56183

**Published:** 2009

**Authors:** R. B. Nerli, Ajay G. Kumar, Ashish Koura, Vikram Prabha, S. B. Alur

**Affiliations:** Department of Urology, KLES Kidney Foundation, Nehru Nagar, Belgaum – 590 010, India

**Keywords:** Quality of life, stress, transobturator, urinary incontinence, vaginal tape

## Abstract

**Background:**

The tension-free vaginal tape (TVT) procedure is based on the integral theory that the midurethra has an important role in the continence mechanism. Transobturator vaginal tape (TOT) is the same in concept as TVT but it differs from TVT in that, rather than passing through the retropubic space, sling materials are drawn through the obturator foramina. We prospectively compared TVT with TOT with respect to operation-related morbidity and surgical outcomes at a minimum follow up of 12 months.

**Materials and Methods:**

A total of 36 women with stress urinary incontinence (SUI) were alternatively assigned to the TVT group (18) or the TOT group. Preoperative evaluation included urodynamic study and I-QOL questionnaire. One year after operation the surgical result, patient satisfaction, incontinence quality-of-life questionnaire, long-term complications, and uroflowmetry were evaluated in both groups.

**Results:**

The patient characteristics in both the TVT and TOT group were similar. Mean operating time was significantly shorter in the TOT group likened to the TVT group.

**Conclusions:**

Both the TVT and TOT procedures are minimally invasive and similar in operation-related morbidity. TOT appears to be as effective as TVT, and safer than TVT for the surgical treatment of SUI in women at 12 months follow-up.

## INTRODUCTION

Until the early 1980s the pathophysiology of stress incontinence was based mainly on Enhorning's theory.[1] The 1980s saw a change in approach inspired by De Lancey's hammock theory.[2] The tension-free vaginal tape (TVT) technique stems from a careful analysis of the physiology of female urinary continence and of the mechanisms possibly causing stress incontinence.[3]

Though various studies have shown significant cure rates with TVT, perioperative complications have been reported, including bowel, vascular, and bladder injuries.[4–7] Most complications are related to blind trocar passage in the retropubic space. To minimize such complications, alternative techniques such as transobturator vaginal tape (TOT) have been developed in which the tape is introduced through the obturator foramen. Delorme *et al*.[8] reported the transobturator technique for a midurethral sling, in which the sling passes the obturator foramen from outside to inside. Reports indicate that TOT provides high short-term cure rates, similar to those achieved with TVT[8]. We prospectively compared the efficacy and safety of TVT and TOT at a minimum follow-up of 12 months.

## MATERIALS AND METHODS

Female patients with urodynamically proven stress urinary incontinence (SUI) were prospectively evaluated as per our departmental protocol during the period January 2003 to December 2006. The protocol included history and physical examination, urinalysis, urine culture, uroflowmetry, postvoid residual (PVR) urine measurement, and multichannel urodynamic studies (MMS UD 2000, Ennschede, The Netherlands). The study inclusion criteria included females, with SUI and age older than 18 years. The study exclusion criteria included the presence of any urinary tract infection, malignancy, pregnancy, severe grade urogenital prolapse (grade III and IV), and predominant urge incontinence. The patients with intrinsic sphincter deficiency (ISD) were part of study group. The severity of urinary incontinence was classified according to the Ingelman-Sundberg scale,[9] as follows: grade 1, urinary incontinence occurs when coughing or sneezing; grade 2, urinary incontinence occurs when running or picking up objects from the floor; and grade 3, urinary incontinence occurs when walking or climbing stairs. The I-QOL questionnaire[10] which evaluates the effect of SUI on patient's everyday life was administered to all the patients.

Patients were alternatively assigned to the TVT or TOT group. We used the TVT technique according to the description of Ulmsten *et al*.[4] and the TOT technique according to the description of Delorme *et al*. of the obturator route of tape insertion[8] with the patient under regional anesthesia with intravenous sedation if required. Cystoscopy was performed only during the TVT procedure. A clamp was kept snugly between sling and suburethral tissue to keep it tension free. On the day of the surgery, duration of the procedure, volume of intraoperative bleeding (blood loss was calculated by weighing blood-stained gauzes), and pain severity after surgery using a visual analog scale were measured. Operative time was calculated from incision start time to closure stop time. Following surgery all patients were catheterized. Peri and postoperative antibiotics were administered. Catheter was removed the next day and the patients underwent studies that included measurements of flow rate and PVR before being discharged home. Postoperative assessment, including pain associated with surgery and time to return to normal activity, was done at one-week and one-month follow-up visits. At a 12-month follow-up visit the patients were evaluated for surgical result, patient satisfaction, I-QOL questionnaire, long-term complications, urinary flow rate, and PVR. The surgical outcomes in the two groups were compared.

Surgical outcome was evaluated by the cough stress test with a comfortably full bladder and symptom questionnaire. The surgical outcome was divided into three groups, including cured, improved, and failed. Patients were considered cured of SUI if they had a negative cough stress test result and there were no reports of urine leakage during stress. Patients were considered to have improved if they had no leakage on the cough stress test but may have had occasional urine leakage during stress. However, this occasional leakage did not influence daily activities or require any further treatment. In patients who did not meet these criteria, treatment was considered to have failed.

## RESULTS

A total of 18 patients underwent the TVT procedure and another 18 underwent the TOT procedure during the study period. The mean age, parity, preoperative clinical parameters, preoperative urodynamic parameters, and mean I-QOL scores were similar in both the groups [Table 1]. The mean ± SD operating time was 21.4 ± 2.75 in the TVT group and 18.4 ± 1.85 minutes in the TOT group [Table 2]. There were no significant differences in the intraoperative bleeding, pain severity after surgery, time to return to normal activity, and PVR between the groups. Following removal of catheter post surgery three patients (16.6%) in the TVT group and two (11.1%) in the TOT group required recatheterization, but all voided well after removing the catheter 48 hours later. One patient in each group complained of pain associated with the operation one week after the surgery, suprapubic discomfort was noted in the patient undergoing TVT and inner thigh discomfort in the patient undergoing TOT. These transient symptoms of discomfort disappeared in all cases at one-month follow-up visit in each group. One patient undergoing TVT had intraoperative bladder perforation. The needle was carefully removed and reinserted under cystoscopic guidance. No other significant perioperative complications such as vaginal injury, retropubic hematoma, bowel injury, or significant nerve and vessel injury were noted in either group.

**Table 1:** Preoperative characteristics of patients who underwent tension-free vaginal tape and transobturator vaginal tape

Characteristics	TVT	TOT
Age (mean ± SD)	49.5 ± 1.95	50.2 ± 1.89
Parity (times) (mean ± SD)	2.3 ± 0.9	2.1 ± 1.01
No. menopause (%)	55	66
Preoperative clinical parameters (mean ± SD)		
Symptom duration (years)	6.6 ± 1.29	6.6 ± 1.29
SUI grade (range 1–3)	1.6 ± 0.5	1.5 ± 0.7
Preoperative urodynamic parameters (mean ± SD):		
MFR (ml/sec)	21.3 ± 2.79	21.8 ± 2.76
PVR (ml)	18.9 ± 12.14	18.3 ± 10.57
Abdominal leak point pressure (cm H_2_O)	92.2 ± 10.1	92.1 ± 8.29
I-QOL scores (mean ± SD)		
Total	56.22 ± 3.47	55 ± 5.31
Avoidance + limiting behavior	40.50 ± 6.46	43.06 ± 6.76
Psychosocial impacts	45.56 ± 8.13	45.33 ± 3.41
Social embarrassment	30.78 ± 6.08	30.94 ± 8.24

**Table 2:** Operation-related complications and morbidity in tension-free vaginal tape versus transobturator vaginal tape

	TVT	TOT	*P* value
Operative time (min) (mean ± SD)	21.4 ± 2.75	18.4 ± 1.85	0.001
Intra-operative bleeding (ml) (mean ± SD)	38.7 ± 5.09	37.2 ± 4.53	NS
Postoperative pain (visual analog scale) (mean ± SD)			
2 hours	2.4 ± 0.45	2.2 ± 0.5	NS
1 week	1.03 ± 0.29	0.95 ± 0.23	NS
1 month	0.16 ± 0.10	0.17 ± 0.09	NS
PVR (ml) (mean ± SD)			
One day postoperation	33.2 ± 16.51	34.2 ± 13.30	NS
One week postoperation	28.6 ± 2.95	27.9 ± 3.8	NS
Postoperative retention (%)	3 (16.6)	2 (11.1)	NS
Days to normal activity (mean ± SD)	4.8 ± 3.2	5.1 ± 3.1	NS
No. operative complications (%)			
Bladder perforations	1 (5.5)	0	NS
Short-term voiding difficulty	3 (16.6)	3 (16.6)	NS
No. postoperative *de novo* urgency (%)	2 (11.1)	3 (16.6)	NS

NS – Not significant			

The surgical results in the two groups were similar [Table 3]. The cure rate was 88.8% in both the groups and so was the improved rate in both the groups. The rate of satisfaction was similar in both the groups and there were no significant differences between the two groups in relation to subjective outcome of the surgery.

**Table 3:** Surgical results in patients with tension-free vaginal tape and transobturator vaginal tape

	No. TVT (%)	No. TOT (%)
SUI (%)		
Cured	16 (88.8)	16 (88.8)
Improved	2 (11.2)	2 (11.2)
Failed	—	—
Subjective satisfaction (%)		
Very satisfied	15 (82.5)	14 (77)
Satisfied	2 (11.1)	3 (16.6)
Not satisfied	1 (5.5)	1 (5.5)
Would undergo sling again if symptoms recur (%)		
Yes	17 (93.5)	17 (93.5)
Not satisfied	1 (5.5)	1 (5.5)
Would recommend sling to other patients (%)		
Yes	17 (93.5)	17 (93.5)
Not satisfied	1 (5.5)	1 (5.5)

**Figure 1:**
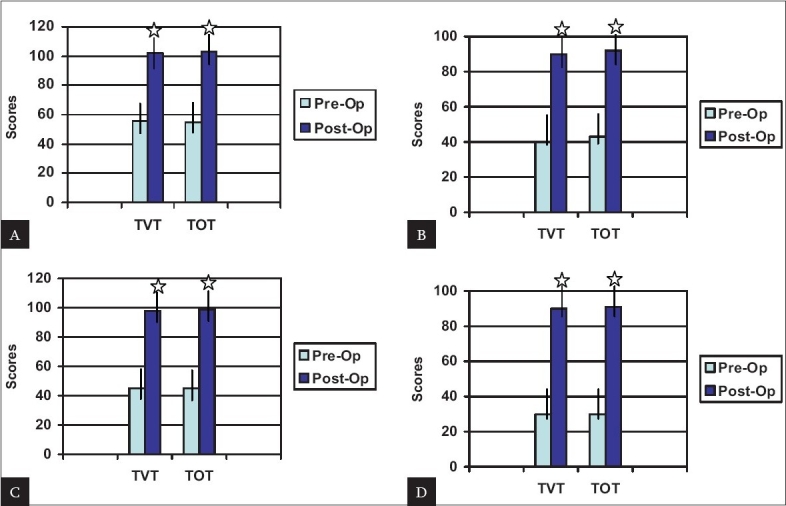
Change in mean I-QOL total and domain scores after TVT and TOT at one-year follow-up. (A) Total scores; (B) Avoidance behaviors; (C) Psychosocial impacts; (D) social embarrassment. Asterisk indicates versus preoperative scores, P < 0.001

At 12-month follow-up there was significant improvement in I-QOL total and domain scores in the two groups [Figures 1A-D]. Mean maximum flow rates (MFRs) decreased significantly in each group postoperatively, that is by 3.4 ml/sec in the TVT group and 3.8 ml/sec in the TOT group. There was no significant difference in the PVR between the groups [Table 4].

**Table 4:** Uroflowmetry and postvoid residual at 12 months in patients with tension-free vaginal tape and transobturator vaginal tape

	Preoperative value (mean ± SD)	Postoperative value (mean ± SD)	*P*-value
MFR (ml/sec)			
TVT	21.3 ± 2.79	17.9 ± 0.90	< 0.05
TOT	21.8 ± 2.76	18 ± 0.91	
PVR (ml)			
TVT	18.9 ± 12.14	20.7 ± 2.91	NS
TOT	18.3 ± 10.57	19.4 ± 1.79	

NS – Not significant			

There was no relation between ALPP and success of surgery. *De novo* urge incontinence symptoms developed postoperatively in two (11.1%) patients in the TVT group and in three (16.6%) in the TOT group. There were no other complications, such as prolonged voiding difficulty, wound infection, or vaginal erosion, in either group.

## DISCUSSION

The TVT sling introduced by Ulmsten *et al*.[6] in 1996 has gained widespread popularity over the last decade supported by long-term prospective data. Delorme[8] in 2001 then described the TOT procedure, which involved the tension-free insertion of a polypropylene tape via a tunneler in a horizontal plane under the midurethra between the two obturator foramina in an ‘outside–in’ orientation. A variation to this technique has been described in 2003 by de LevalIntrinsic sphincter deficiency[11] termed the TOT vaginal tape ‘inside–out’ technique which directs the needles in the opposite orientation.

Tension-free vaginal tapes have revolutionized the treatment of female SUI.[4] While TVT have proven to have long-term efficacy,[5] their introduction via a retropubic route has been associated with a number of perioperative or postoperative complications resulting from penetration of the tape or supporting needle in bladder, urethra, bowel, nerves, or vessels.[12] Abouasally *et al*.[13] analyzed the complications of TVT procedure at six institutions. Complications during the procedure included bladder perforation in 48 patients (5.8%) and blood loss >500 ml in 16 (2.5%). Immediate complications after surgery were urinary retention (>24 hours after) in 47 patients (19.7%), pelvic hematoma in four (1.9%), and suprapubic wound infection in one (0.4%). Of the 47 patients in retention, 32 were in retention for < 48 hours and treated with an indwelling catheter. The 15 remaining patients were treated with an indwelling catheter (one) or clean intermittent catheterization for a mean of 22 days. To correct the retention the TVT was released in seven patients and the tape sectioned in three. Late complications were *de novo* urgency, persistent suprapubic discomfort, and intravaginal tape erosion in 36 (15%), 18 (7.5%), and 1 (0.4%) patient, respectively. Most of these complications resolved with observation and medical treatment, but intravaginal tape erosion required partial resection of the tape with closure and repair of the vaginal mucosa.

In 2001 transobturator approach was proposed for the placement of suburethral tapes with the aim of sparing the retropubic space[8]. Initial clinical data[8] as well as results of cadaver dissections[14] suggested that complication rates may be decreased by this approach. Studies evaluating the efficacy of the TOT approach have been relatively short-term but show promising results. Cure rates have ranged from 80.5–97% on the basis of objective testing and subjective questionnaires or quality of life instruments.[14,15,17,21] A study by Chen *et al*.[16] in 54 patients found an 85% urodynamic cure with an additional improvement rate with the TOT. In a study of 120 patients who underwent TOT, Roumeguere *et al*.[17] had 31 subjects with maximum urethral closure pressures (MUCPs) of 30 cm water or less. Cure rates were noted to be 86% if MUCPs were greater than 30 cm water, compared to 81% if MUCP was between 20 and 30 cm water and 70% if MUCP was less than 20 cm water. Larger, long-term studies in these subsets of patients are required.

Tension-free vaginal tape and TOT have been compared in randomized controlled trials (RCTs) for treatment of SUI. De Tayrac *et al*.[18] prospectively compared in a randomized fashion TVT and TOT for surgical treatment of SUI. Patient characteristics, preoperative QOL, and urodynamic evaluation were similar in the two groups. Mean operative time was significantly shorter in the TOT group. No bladder injury occurred in the TOT group versus 9.7% in the TVT group. The rate of postoperative urinary retention was 25.8% in the TVT group versus 13.3% in the TOT group. The rates of cure (83.9 vs. 90%), improvement (9.7 vs. 3.3%), and failure (6.5 vs. 6.7%) were similar for the TVT and TOT groups, respectively. They concluded that TOT was equally efficient as TVT for surgical treatment of SUI in women, with no reduction of bladder outlet obstruction at one-year follow-up.

Similarly Lee *et al*.[19] prospectively compared the efficacy and safety of TVT and TOT inside–out for female SUI. A total of 120 women with SUI were alternatively assigned to the TVT group or the TOT group. Patient characteristics were comparable in both groups. Mean operating time was significantly shorter in the TOT group. The rates of cure (86.8 vs. 86.8%), improvement (6.6 vs. 8.2%), and failure (6.6 vs. 5%) were similar in TOT and TVT groups, respectively. I-QOL questionnaire parameters one year after surgery were improved significantly in each group and there was no difference between the groups. There were no long-term complications in either group. *De novo* urgency developed in four patients in the TOT group. They concluded that TVT and TOT were minimally invasive and similar in operation-related morbidity. TOT appeared to be as effective and safe as TVT for the surgical treatment of SUI in women at one-year follow-up.

There are no published RCTs comparing the TOT outside–in and inside–out techniques. Debodinance[20] presented a nonrandomized prospective, observational study of 100 patients at 12 months follow-up, half of whom received TOT outside–in and the other half, TOT inside–out. There were no differences in patient characteristics of the either group with respect to mixed incontinence, previous incontinence surgery, or incidence of intrinsic sphincter deficiency. Cure rates at one year were 94 versus 90% (not statistically significant), respectively.

There is a paucity of data in the literature to direct physicians as to the best indications for the use of the various midurethral slings. William Andre Silva[21] reviewing the various midurethral slings recommended retropubic midurethral sling in the following situations: (1) younger patients, (2) intrinsic sphincter deficiency (Rezapour *et al*.[22] reported an 86% rate of cure or improvement over four years with TVT), and (3) physically active patients (avoids thigh/groin discomfort with exercise). He also suggested that a TOT sling may be preferred over a retropubic sling in the following situations: (1) obesity (reduced risk or difficulty in passage of needles), (2) previous retropubic or major abdominal surgery, (3) elderly, and (4) mixed incontinence. There are no conclusive data to recommend one over the other. Usually, surgeon preference and training will play a role in decision making.

## CONCLUSIONS

The concept of the midurethral sling has revolutionized surgical treatment of SUI. Its minimally invasive approach and success rates have led to an increasing acceptance of the technique. In short-term follow-up TOT appears to be as effective as TVT, shorter operating time than TVT and has fewer complications. TOT is safe and effective in women with SUI.
